# Development and Validation of a Multivariable Predictive Model for Mortality of COVID-19 Patients Demanding High Oxygen Flow at Admission to ICU: AIDA Score

**DOI:** 10.1155/2021/6654388

**Published:** 2021-06-30

**Authors:** Marija Zdravkovic, Viseslav Popadic, Slobodan Klasnja, Vedrana Pavlovic, Aleksandra Aleksic, Marija Milenkovic, Bogdan Crnokrak, Bela Balint, Milena Todorovic-Balint, Davor Mrda, Darko Zdravkovic, Borislav Toskovic, Marija Brankovic, Olivera Markovic, Jelica Bjekic-Macut, Predrag Djuran, Lidija Memon, Ana Stojanovic, Milica Brajkovic, Zoran Todorovic, Jovan Hadzi-Djokic, Igor Jovanovic, Dejan Nikolic, Dane Cvijanovic, Natasa Milic

**Affiliations:** ^1^University Clinical Hospital Center, Bezanijska kosa, Belgrade, Serbia; ^2^Faculty of Medicine, University of Belgrade, Belgrade, Serbia; ^3^Institute for Medical Statistics and Informatics, Faculty of Medicine University of Belgrade, Belgrade, Serbia; ^4^Clinical Center of Serbia, Belgrade, Serbia; ^5^Institute of Cardiovascular Diseases “Dedinje”, Belgrade, Serbia; ^6^Department of Medical Sciences, Serbian Academy of Sciences and Arts, Serbia; ^7^Clinic for Hematology, Clinical Center of Serbia, Belgrade, Serbia; ^8^University Clinical Center Zvezdara, Belgrade, Serbia; ^9^Department of Internal Medicine, Division of Nephrology and Hypertension, Mayo Clinic, Rochester, USA

## Abstract

**Introduction:**

Risk stratification is an important aspect of COVID-19 management, especially in patients admitted to ICU as it can provide more useful consumption of health resources, as well as prioritize critical care services in situations of overwhelming number of patients.

**Materials and Methods:**

A multivariable predictive model for mortality was developed using data solely from a derivation cohort of 160 COVID-19 patients with moderate to severe ARDS admitted to ICU. The regression coefficients from the final multivariate model of the derivation study were used to assign points for the risk model, consisted of all significant variables from the multivariate analysis and age as a known risk factor for COVID-19 patient mortality. The newly developed AIDA score was arrived at by assigning 5 points for serum albumin and 1 point for IL-6, D dimer, and age. The score was further validated on a cohort of 304 patients admitted to ICU due to the severe form of COVID-19.

**Results:**

The study population included 160 COVID-19 patients admitted to ICU in the derivation and 304 in the validation cohort. The mean patient age was 66.7 years (range, 20–93 years), with 68.1% men and 31.9% women. Most patients (76.8%) had comorbidities with hypertension (67.7%), diabetes (31.7), and coronary artery disease (19.3) as the most frequent. A total of 316 patients (68.3%) were treated with mechanical ventilation. Ninety-six (60.0%) in the derivation cohort and 221 (72.7%) patients in the validation cohort had a lethal outcome. The population was divided into the following risk categories for mortality based on the risk model score: low risk (score 0–1) and at-risk (score > 1). In addition, patients were considered at high risk with a risk score > 2. By applying the risk model to the validation cohort (*n* = 304), the positive predictive value was 78.8% (95% CI 75.5% to 81.8%); the negative predictive value was 46.6% (95% CI 37.3% to 56.2%); the sensitivity was 82.4% (95% CI 76.7% to 87.1%), and the specificity was 41.0% (95% CI 30.3% to 52.3%). The *C* statistic was 0.863 (95% CI 0.805-0.921) and 0.665 (95% CI 0.598-0.732) in the derivation and validation cohorts, respectively, indicating a high discriminative value of the proposed score.

**Conclusion:**

In the present study, AIDA score showed a valuable significance in estimating the mortality risk in patients with the severe form of COVID-19 disease at admission to ICU. Further external validation on a larger group of patients is needed to provide more insights into the utility of this score in everyday practice.

## 1. Introduction

COVID-19 infection represents a highly contagious infective disease with a wide array of clinical presentations and a massive burden for the health systems worldwide [1–4]. Symptoms at disease onset are relatively mild, and a significant group of patients does not show apparent symptoms before the development of respiratory failure, which makes it more difficult to identify patients at risk [5–7]. Different prediction models were developed based on various demographic, radiographic, and laboratory parameters but only a few of them focusing on clinical risk, ICU care, and in-hospital mortality [8–10]. Patients with the severe form of the disease were more likely to be older, associated with multiple comorbidities, severe lung involvement, and immune response [11, 12].

Risk stratification is a very important part of the management of COVID-19 mostly due to the need to prioritize critical care services in situations of an overwhelming number of patients. A proper risk stratification could provide more useful consumption of health resources, as well as to reorient more attention to the patients most likely to develop a severe form of the disease [13–15]. In certain studies, it is shown that predictive models using laboratory parameters had stronger discriminatory power compared to the clinical models [16]. Careful monitoring of laboratory and clinical parameters followed by a purposeful risk stratification of patients admitted to ICU could allow a forehand reaction in case of disease progression, reducing further deterioration and overall mortality.

In this multicenter study, we aimed to develop and validate a multivariable predictive model for mortality of COVID-19 patients admitted to ICU.

## 2. Materials and Methods

The AIDA score was developed according to the results and methodology of the previous study by Popadic et al. [17], combining all significant variables from the multivariate logistic regression analysis including serum albumin, interleukin-6, and D-dimer, accompanied by age.

### 2.1. Study Population and Risk Factors

The derivation group consisted of 160 COVID-19 patients with moderate to severe ARDS admitted to the Respiratory Intensive Care Unit between June 23, 2020, and October 2, 2020, in University Clinical Hospital Center Bezanijska kosa, Belgrade, Serbia, while further analysis and validation were performed on additional consecutive 318 patients admitted to ICU between October 2, 2020, and January 14, 2021, in University Clinical Hospital Center Bezanijska kosa, Belgrade, Serbia (160 patients), and University Clinical Hospital Center Zvezdara, Belgrade, Serbia (158 patients). The patients in both groups were treated by the National Protocol of the Republic of Serbia for the treatment of COVID-19 infection, as explained in Materials and Methods of the development study by Popadic et al. [17].

The Institutional Review Boards of the University Clinical Hospital Center Bezanijska kosa and University Clinical Hospital Center Zvezdara approved the conducting of the study.

### 2.2. Predictive Model Development

The predictive model was developed using data completely from a development cohort, which consisted of 160 patients. Patient characteristics were first assessed by univariate logistic regression analysis, following with the final model being developed using a stepwise multivariate logistic regression analysis. The characteristics pool for stepwise-regression modeling was defined based on characteristics known relevance or correlation with increased mortality risk (*p* value < 0.10 in univariate analysis). The variance inflation factor (VIF) was used to examine covariates for colinearity. The risk prediction score was developed using coefficients from the final regression multivariate model with the addition of age from univariate analysis. Missing data was rare (<5%) among characteristics considered for the final model development, and no imputations were performed. Patients who had their data missing for an outcome (14 patients in total) were excluded from the analysis. Wilson procedure, including continuity correction, was used to evaluate differences between characteristics frequency in the development and validation cohorts, shown with a 95% confidence intervals (CI). Patients were divided into 2 risk groups according to the risk score once the final model had been defined.

### 2.3. Model Validation

The validation cohort (304 patients) was used to assess the final model. Definitions, measurements, and outcomes used in the validation study were the same as the ones used in the development study. Model discrimination performance was tested by means of sensitivity, specificity, positive, and negative predictive values. *C* statistic, representing the area under the receiver operating characteristic curve, was used for overall assessment of the predictive model. Larger values of *C* statistics indicated improved discrimination. For the statistical analysis, the SPSS version 25 statistical software (Chicago, USA) was used.

## 3. Results

### 3.1. Patient Characteristics

The study population included 160 COVID-19 patients admitted to ICU in the derivation and 304 in the validation cohort. The two cohorts were well balanced concerning the most assessed patient characteristics (Table 1). The mean patient age was 66.7 years (range, 20–93 years), with 68.1% men and 31.9% women. Most patients (76.8%) had comorbidities with hypertension (67.7%), diabetes (31.7), and coronary artery disease (19.3) as the most frequent. Obesity was present in 16.5% of patients and was more prevalent in the validation cohort. A total of 316 patients (68.3%) were treated with mechanical ventilation, and 89 (19.2%) received Tocilizumab. Ninety-six (60.0%) in the derivation cohort and 221 (72.7%) patients in the validation cohort had a lethal outcome.

### 3.2. Risk Assessment Model

In the derivation cohort, the following variables were associated with the mortality of patients admitted to ICU due to COVID-19-related pneumonia in univariate logistic regression analysis: age (RR = 3.495, 95% CI 1.801–6.779), albumin (RR = 22.286, 95% CI 9.319–53.294), D-dimer (RR = 2.111, 95% CI 1.091–4.085), IL-6 at admission to ICU (RR = 6.100, 95% CI 2.857–13.023), and CT score (RR = 2.362, 95% CI 1.120–4.980). In the multivariate analysis, serum albumin (RR = 25.740, 95% CI 7.491–88.443), IL-6 (RR = 6.245, 95% CI 1.937–20.129), and D-dimer at admission to ICU (RR = 4.574, 95% CI 1.375–15.212) were independently associated with mortality [17]. Subsequently, the regression coefficients from the final multivariate model were used to assign points for the risk model. The newly developed AIDA score included all significant patient characteristics from the multivariate analysis and age as a known risk factor for COVID-19 patient mortality. The AIDA score was arrived at by assigning 5 points for serum albumin and 1 point for IL-6, D dimer, and age (Table 2). Finally, based on the risk prediction score, the population consisted of the following division risk categories for mortality: low risk (score 0–1) and at risk (score > 1). In addition, patients were considered at high risk with a risk score > 2.

### 3.3. Accuracy and Validation of AIDA Risk Model

In the development cohort, for patients classified as at risk (score > 1), the AIDA risk model produced a positive predictive value (probability of a lethal outcome in patient designated at risk) of 73.8% (95% CI 68.9% to 78.2%) and a negative predictive value (probability of recovering in patients designated low risk) of 91.2% (95% CI 76.7% to 97.0%). The sensitivity (probability of being classified as at risk in patients with the lethal outcome) was 96.9% (95% CI 91.1% to 99.4%), and the specificity (probability of being classified as low risk in patients recovered) was 48.4% (95% CI 35.8% to 61.3%). A high-risk AIDA score > 2 had a positive predictive value (probability of a lethal outcome in patient designated at high risk) of 81.2% (95% CI 74.6% to 86.4%) and a negative predictive value (probability of recovering in patients designated as not being at high risk) of 76.3% (95% CI 65.9% to 84.3%). The sensitivity (probability of being classified as at high risk in patients with the lethal outcome) was 85.4% (95% CI 76.7% to 91.8%), and the specificity (probability of being classified as not at high risk in patients recovered) was 70.3% (95% CI 57.6% to 81.1%).

**Figure 1:**
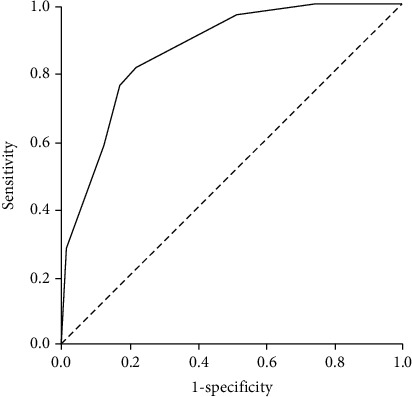
ROC curve in development cohort.

**Figure 2:**
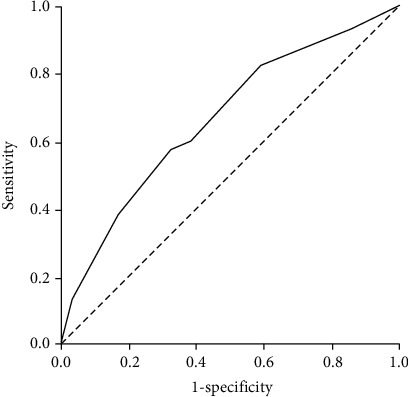
ROC curve in validation cohort.

The AIDA risk model was then tested for accuracy in the validation cohort (*n* = 304), where the positive predictive value was 78.8% (95% CI 75.5% to 81.8%); the negative predictive value was 46.6% (95% CI 37.3% to 56.2%); the sensitivity was 82.4% (95% CI 76.7% to 87.1%), and the specificity was 41.0% (95% CI 30.3% to 52.3%). The *C* statistic was 0.863 (95% CI 0.805-0.921) and 0.665 (95% CI 0.598-0.732) in the development (Figure 1) and validation cohorts (Figure 2), respectively. Both cohorts were similar according to AIDA score accuracy, as well as the frequency of patients classified into each risk category.

## 4. Discussion

The clinical setting of COVID-19 infection could be diverse, affecting multiple organs and provoking various symptoms and signs, making it more difficult to enable an appropriate risk stratification of these patients [18]. Also, the clinical course of the disease in terms of different pathophysiological mechanisms and complications, including acute respiratory distress syndrome, superinfection, shock, acute heart, liver, and kidney injury, is unpredictable and is leaving a limited timespan to bring the right treatment decision in a real clinical scenario [19, 20].

In the present study, we reported a process of development and validation of a multivariable predictive model for mortality of COVID-19 patients demanding high oxygen flow at admission to ICU.

In order to develop a simple but highly predictable risk score, we have started by identifying credible predictors of mortality in a group of patients with the worst possible clinical condition, considering respiratory status as the most important aspect. This is why the derivation group consisted only of patients with moderate to severe ARDS and on invasive, noninvasive mechanical ventilation and high flow oxygen therapy. The main aim of the following analysis was to extract only those clinical and laboratory parameters which are most likely to be linked with the poor clinical outcome. In the final multivariate analysis, serum albumin, interleukin-6, and D-dimer, accompanied by age and CT severity score as parts of univariate analysis, were marked as independent predictors of mortality. It is important to underline that these predictors are reflecting the three most probable pathophysiological mechanisms of a lethal outcome, infection with sepsis and shock, procoagulable state provoking micro and macrothrombosis, and cytokine storm as a potential trigger of multiorgan failure [21]. Different risk scores have been developed to stratify hospitalized COVID-19 patients, with very few being applicable in patients admitted to ICU. AIDA score primarily relies on the high sensitivity (being 82.4% (95% CI 76.7% to 87.1%) in the validation group) and positive predictive value (78.8% (95% CI 75.5% to 81.8%)), as the most important part of the risk stratification in COVID-19 patients was to identify patients at risk, but not to eliminate the subgroup of low-risk patients, as that would be a two-edged sword, considering the unpredictability of the disease and rapid progression of certain clinical forms. One of the most important advantages of this score is a quite respectable sample size in both derivation and validation groups, encompassing more than 460 patients admitted to ICU with a severe form of the disease. The validation group consisted of patients from two different hospital centers but treated according to the same therapy protocol, while the baseline characteristics between the derivation and validation group did not differ significantly (Table 1). The score is easy to use, as it includes usual laboratory parameters for every COVID-19 patient. Also, the significance of CT severity score was already marked as an important part of risk stratification, although it was not statistically analyzed in our study, due to the lack of data in the external validation group. However, it can be helpful as an additional factor considering the results of the univariate logistic regression model where values of CT severity score above 20 were highly predictable for poor clinical outcome among patients admitted to ICU.

Interleukin-6 values above 72 pg/mL were significant for predicting poor clinical outcomes, which may be helpful in the decision-making process, as immunomodulatory therapy should be administrated earlier in the clinical course. According to the results, the interleukin-6 receptor antagonists might be effective in patients with elevated values of interleukin-6 (but below 72 pg/mL), and before the clinical deterioration in terms of respiratory failure and need for mechanical ventilation, as positive results in terms of lower mortality rate among this subset of patients are still to be demonstrated [22]. As presented in our derivation study, the mortality rates did not differ between the groups that received and did not receive Tocilizumab in ICU, although the baseline characteristics were not significantly different.

It is of great importance to note that a low value of serum albumin, below 33 g/L, is already highly significant as a predictor of mortality and is a sufficient parameter to stratify patients into a high-risk group. Following the worsening of the patient's condition, the values of interleukin-6 and D-dimer were usually already elevated above their significant values, while the value of serum albumin was preserved a certain amount of time before further clinical worsening. In patients with a severe form of COVID-19, hypoalbuminemia should be considered the most pertinent marker of an advanced clinical condition and is usually followed by the further rising of proinflammatory parameters and D-dimer, indicating a coupling of progressed shock and an increased procoagulable state [23]. This is meaningful as it can point out that various important regulatory mechanisms are already expended, initiating an irreversible condition refractory to a wide specter of different therapeutic modalities [24]. The potential therapeutical benefit of albumins in patients with COVID-19 is yet to be established [25].

The main limitation of the study is a lack of a more comprehensive external validation in a condition of different therapy protocols being used. It is still unknown if different therapeutic modalities in the earlier phase of the disease can significantly affect the credibility of the score, although this score is primarily intended for the risk stratification of patients admitted to ICU. The score could be developed further by implementing different ICU scoring systems to encompass a wider image of the patient's current condition.

## 5. Conclusion

Risk stratification of patients with COVID-19 is an important aspect of everyday practice, having in mind the unpredictability of clinical course and possible complications of the disease. AIDA score could be a reliable tool capable of identifying patients with a higher risk of poor clinical outcomes at admission to the ICU, providing more space to deliver an appropriate therapy on time. Further validation on a larger group of patients will provide more insights into the utility and definitive clinical significance of this score.

## Figures and Tables

**Table 1:** Characteristics of patients in both development and validation cohorts.

Patient characteristics	Development cohort (*n* = 160)	Validation cohort (*n* = 304)	Difference	95% CI for the difference
*Gender,n(%)*				
Male	110 (68.8)	206 (67.8)	-0.010	-0.100 to 0.080
Female	50 (31.3)	98 (32.2)		
Age, mean ± sd	65.6 ± 14.0	67.2 ± 12.6	-1.559	-4.067 to 0.949
CT score, mean ± sd	19.0 ± 4.9	17.6 ± 6.0	1.394	0.009 to 2.778
Mechanic ventilation, *n* (%)	107 (66.9)	209 (69.0)	-0.021	-0.111 to 0.069
*Comorbidities,n(%)*				
Hypertension	109 (69.4)	203 (66.8)	0.027	-0.064 to 0.117
Diabetes	52 (33.1)	94 (31.0)	0.021	-0.069 to 0.111
Obesity	14 (8.9)	62 (20.4)	-0.115	-0.168 to -0.044
HOBP	8 (5.1)	13 (4.3)	0.008	-0.032 to 0.049
Asthma	6 (3.8)	14 (4.6)	-0.008	-0.047 to 0.032
Coronary disease	28 (17.8)	61 (20.1)	-0.022	-0.099 to 0.054
Cardiomyopathy	14 (8.9)	24 (7.9)	0.010	-0.043 to 0.063
Total number of patients with comorbidities, *n* (%)	120 (75.9)	235 (77.3)	-0.014	-0.095 to 0.068
Total number of patients with 2+ comorbidities, *n* (%)	72 (45.6)	137 (45.1)	0.005	-0.091 to 0.101
Tocilizumab, *n* (%)	38 (23.8)	51 (16.8)	0.070	-0.006 to 0.145

**Table 2:** Predictive model for mortality in patientss admitted to ICU due to COVID-19-related pneumonia.

Variable	Assigned score
Albumin, serum < 33 g/L	5
IL6 > 72 pg/mL	1
D dimer > 1000 ng/mL	1
Age ≥ 65 years	1

## Data Availability

The data that support the findings of this study are available from the corresponding author (MZ) upon reasonable request.

## Abbreviations

COVID-19: Coronavirus disease 19
ICU: Intensive care unit
ARDS: Acute respiratory distress syndrome
CT: Computerized tomography
IL-6: Interleukin-6.

## References

[1] Sarkesh A., Daei Sorkhabi A., Sheykhsaran E. (2020). Extrapulmonary clinical manifestations in COVID-19 patients. *The American Journal of Tropical Medicine and Hygiene*.

[2] Bandyopadhyay D., Akhtar T., Hajra A. (2020). COVID-19 pandemic: cardiovascular complications and future implications. *American Journal of Cardiovascular Drugs*.

[3] Lai C. C., Ko W. C., Lee P. I., Jean S. S., Hsueh P. R. (2020). Extra-respiratory manifestations of COVID-19. *International Journal of Antimicrobial Agents*.

[4] Kordzadeh-Kermani E., Khalili H., Karimzadeh I. (2020). Pathogenesis, clinical manifestations and complications of coronavirus disease 2019 (COVID-19). *Future Microbiology*.

[5] George P. M., Barratt S. L., Condliffe R. (2020). Respiratory follow-up of patients with COVID-19 pneumonia. *Thorax*.

[6] Ottestad W., Søvik S. (2020). COVID-19 patients with respiratory failure: what can we learn from aviation medicine?. *British Journal of Anaesthesia*.

[7] Serafim R. B., Póvoa P., Souza-Dantas V., Kalil A. C., Salluh J. I. F. (2021). Clinical course and outcomes of critically ill patients with COVID-19 infection: a systematic review. *Clinical Microbiology and Infection*.

[8] Wynants L., van Calster B., Collins G. S. (2020). Prediction models for diagnosis and prognosis of COVID-19: systematic review and critical appraisal. *BMJ*.

[9] Parohan M., Yaghoubi S., Seraji A., Javanbakht M. H., Sarraf P., Djalali M. (2020). Risk factors for mortality in patients with coronavirus disease 2019 (COVID-19) infection: a systematic review and meta-analysis of observational studies. *The Aging Male*.

[10] Leeuwenberg A. M., Schuit E. (2020). Prediction models for COVID-19 clinical decision making. *The Lancet Digital Health*.

[11] Armstrong R. A., Kane A. D., Cook T. M. (2020). Outcomes from intensive care in patients with COVID-19: a systematic review and meta-analysis of observational studies. *Anaesthesia*.

[12] Mudatsir M., Fajar J. K., Wulandari L. (2020). Predictors of COVID-19 severity: a systematic review and meta-analysis. *F1000Research*.

[13] Cherian R. (2021). Clinical risk stratification in COVID-19: the need for a revised approach?. *Pulmonary Circulation*.

[14] Smilowitz N. R., Nguy V., Aphinyanaphongs Y. (2021). Multiple biomarker approach to risk stratification in COVID-19. *Circulation*.

[15] Xu R., Hou K., Zhang K. (2020). Performance of two risk-stratification models in hospitalized patients with coronavirus disease. *Frontiers in Medicine*.

[16] Henry B. M., De Oliveira M. H., Benoit S., Plebani M., Lippi G. (2020). Hematologic, biochemical and immune biomarker abnormalities associated with severe illness and mortality in coronavirus disease 2019 (COVID-19): a meta-analysis. *Clinical Chemistry and Laboratory Medicine*.

[17] Popadic V., Klasnja S., Milic N. (2021). Predictors of mortality in critically ill COVID-19 patients demanding high oxygen flow: a thin line between inflammation, cytokine storm, and coagulopathy. *Oxidative Medicine and Cellular Longevity*.

[18] da Rosa Mesquita R., Francelino Silva Junior L. C., Santos Santana F. M. (2021). Clinical manifestations of COVID-19 in the general population: systematic review. *Wiener Klinische Wochenschrift*.

[19] Wang Z., Deng H., Ou C. (2020). Clinical symptoms, comorbidities and complications in severe and non-severe patients with COVID-19: a systematic review and meta-analysis without cases duplication. *Medicine (Baltimore)*.

[20] Vakili K., Fathi M., Pezeshgi A. (2020). Critical complications of COVID-19: a descriptive meta-analysis study. *Reviews in Cardiovascular Medicine*.

[21] Jose J. R., Manuel A. (2020). COVID-19 cytokine storm: the interplay between inflammation and coagulation. *The Lancet Respiratory Medicine*.

[22] Du P., Geng J., Wang F., Chen X., Huang Z., Wang Y. (2021). Role of IL-6 inhibitor in treatment of COVID-19-related cytokine release syndrome. *International Journal of Medical Sciences*.

[23] Ramadori G. (2020). Hypoalbuminemia: an underestimated, vital characteristic of hospitalized COVID-19 positive patients?. *Hepatoma Research*.

[24] Lee C., Choi W. J. (2021). Overview of COVID-19 inflammatory pathogenesis from the therapeutic perspective. *Archives of Pharmacal Research*.

[25] Violi F., Ceccarelli G., Loffredo L. (2021). Albumin supplementation dampens hypercoagulability in COVID-19: a preliminary report. *Thrombosis and Haemostasis*.

